# A randomised, double-blind clinical study into the effect of zinc citrate trihydrate toothpaste on oral plaque microbiome ecology and function

**DOI:** 10.1038/s41598-025-92545-0

**Published:** 2025-03-08

**Authors:** Suzi Elizabeth Adams, Andrew Kenneth Cawley, David Arnold, Michael John Hoptroff, Vera Slomka, Jane Reid Matheson, Robert Edward Marriott, Matthew Ronald Gemmell, Philip David Marsh

**Affiliations:** 1 Unilever Oral Care, Bromborough Road, Bebington, CH63 3JW UK; 2 Institute of Integrative Biology, Centre for Genomic Research, University of Liverpool, Crown Street, Liverpool, L69 7ZB UK; 3 Department of Oral Biology, School of Dentistry, University of Leeds, Leeds, LS2 7TF UK

**Keywords:** Clinical microbiology, Microbial communities

## Abstract

The oral microbiome is a diverse community of microbes residing on all oral surfaces. A balanced oral microbiome is associated with good oral health, and disruption can result in imbalance associated with diseases including gingivitis and dental caries. It is important, therefore, to understand how daily use of oral hygiene products impacts the microbiome. Composition and activity of dental plaque microbiome from 115 participants was analysed after brushing with one of two toothpastes, one containing zinc citrate trihydrate and the other a control toothpaste, in a parallel design. Each participant brushed twice daily for 6-weeks, with samples collected at baseline, 2 and 6-weeks. Metataxonomic analysis demonstrated changes in bacterial communities with use of the zinc toothpaste compared to the control product at community and species level. Increases at the species level were observed for taxa from the genus *Veillonella* with decrease in a taxon from the genus *Fusobacterium* for the zinc toothpaste. Analysis of microbiome function based on predicted metagenomic and metatranscriptomic analysis show that use of the zinc toothpaste is associated with an in-vivo reduction in glycolysis, consistent with the mode of action of zinc and, increases in processes linked to gum-health (lysine biosynthesis), and to whole-body health (nitrate reduction). Our findings provide the first understanding of the beneficial modulation of microbiome composition and function by zinc-containing toothpaste in-vivo for oral care benefits.

**Supplementary Information:**

The online version contains supplementary material available at 10.1038/s41598-025-92545-0.

## Introduction

The study of oral bacterial communities and their response to the use of toothpaste has focused on bacterial viability^1–3^ and reduction in dental biofilm load alongside clinical parameters for gingivitis such as gingival inflammation and bleeding^4,5^. It is important to reduce the levels of plaque bacteria through regular oral hygiene to mitigate the risk of oral conditions such as gingivitis and dental caries^6–8^. It is increasingly recognised that maintaining an appropriately balanced, health-associated bacterial community in the regrowing dental biofilm is essential and that active agents present in toothpaste formulations can aid this process^8,9^.

Standard microbiology techniques alone cannot build a detailed picture of bacteria present in the regrowing plaque due to the inability to culture and enumerate all species present^10^. However, continued development of molecular techniques allows comprehensive taxonomic and functional analysis of the oral microbiome to study the effects of active toothpaste ingredients, on that community. Previously, it has been demonstrated using metataxonomics, that the microbiome of dental biofilms can be shifted to a healthier state with regular use of toothpaste containing enzymes and proteins^11^.

Metataxonomics permits characterisation of the oral microbiota, its constituent taxa, their relative abundance within the community and overall diversity of that community^12^. The approach can be augmented with functional prediction tools such as Tax4Fun^13^ to provide insight into the metabolic potential of the community. Understanding both taxonomic and functional output is important, as changes in the taxonomic composition may not generate changes in the microbiome function and vice versa due to the high level of functional redundancy between bacterial species^14,15^. Combining these approaches can provide a rich and informative dataset^16^. In addition, the use of shotgun metagenomic or metatranscriptomic approaches can provide a more detailed measurement-based interpretation of the real in-situ metabolic potential and activity of the microbial community and the impact of toothpaste use.

Toothpaste formulations containing zinc salts have been used for over 30 years^4,10,17^. Zinc salts are bacteriostatic, reducing biofilm regrowth and inhibiting enzymes involved in bacterial processes such as glycolysis, postprandial acid production and oral malodour production^17–20^. These actives have been studied in multiple clinical trials and in-vitro experiments to understand the mode of action of zinc salts against a range of bacteria^21,22^. However, little is known regarding the influence of zinc salts on oral microbial composition and metabolic activity and the changes that occur with regular brushing with a zinc containing toothpaste compared to a control toothpaste. The work presented here describes for the first time such a study where volunteers used either a zinc citrate trihydrate toothpaste or a control toothpaste, with supragingival plaque samples collected at baseline, and after 2 and 6 weeks of product use, for microbiome analysis. Individual samples were studied using 16S rRNA metataxonomic analysis to examine community composition changes using beta diversity alongside changes in individual taxa. Taxa were classified at species level, which is essential due to the presence of species associated with oral health and disease within the same genera. For example, in the genus *Streptococcus*,* S. sanguinis* is associated with oral health and *S. mutans* with dental caries^23^.

Although it is useful to recognise changes in relative abundance of individual bacteria following the use of toothpaste, it is also important to understand changes in functional output based on the community as a whole^15^. Computational prediction of functional output was employed to examine changes in bacterial metabolism with toothpaste use, both over time and between zinc and control toothpastes. Metatranscriptomic analysis was conducted to determine the changes at community level on individual genes and genes grouped by pathway based on time point and toothpaste. The ability to study changes in functional output of the bacterial community, at the metabolic and genetic level, can give greater insight into the mode of action of toothpaste formulations.

The aim of this study was to examine the effect of a zinc citrate trihydrate toothpaste (zinc toothpaste) on the dental biofilm at both taxonomic and functional level compared to a control toothpaste. The combination of microbiome techniques allows for a more thorough picture of gene level changes in the functional profile of the whole microbiome.

## Results

### Clinical results

**Fig. 1:**
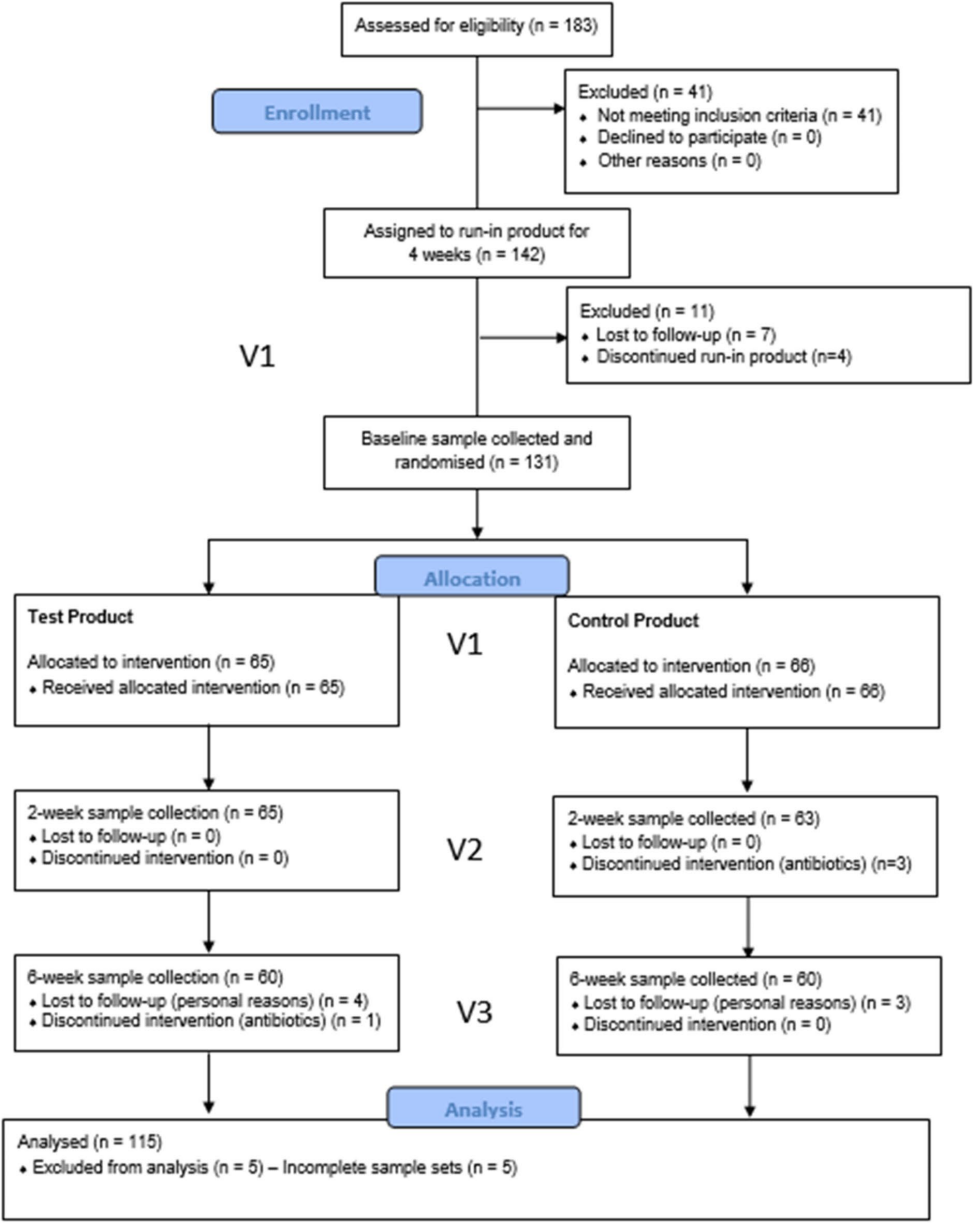
CONSORT 2010 flow diagram.

The mean age of subjects was 43, with 25 male and 106 female participants randomised to either zinc or control toothpaste (Table 1; Fig. 1).

**Table 1:** Study demographics.

Study phase	Test		Control			Test		Control	
	Number		Number			Age		Age	
	Male	Female	Male	Female		Male	Female	Male	Female
Baseline and Randomisation (131)	10	55	15	51	Average	41	44	43	42
					SD	10	9	8	10
					Range	20–55	22–64	28–60	21–59
Analysed (115)	9	50	13	43	Average	40	43	44	43
					SD	10	9	9	9
					Range	20–55	22–64	28–60	23–59

No toothpaste related adverse events were reported during the study.

### Metataxonomic results

Three hundred and forty-five plaque samples along with six positive DNA mock community controls and six negative buffer controls were processed and analysed via Illumina sequencing, generating over 48 million reads resulting in 908 species-level taxa. Taxa with counts of fewer than 100 reads were aggregated, leaving 584 species-level taxa for statistical analysis. Two samples with fewer than 20,000 reads were removed at this stage, with 343 samples remaining. Positive and negative controls were run as part of each batch of samples, mock community composition was consistent between batches (Supplementary Fig. S1) and no reads were found in the negative controls.

Analysis was carried out at species-level to determine the effect of use of the toothpastes over the 6-week period. Analysis at the community level was conducted using ANOVA to compare the groups; this revealed significant changes associated with both time and toothpaste. No significant difference was observed between toothpastes at baseline (*p* = 0.607) or at 2-weeks (*p* = 0.158), however a significant difference was observed between toothpaste groups after 6-weeks use (*p* = 0.032). On comparing communities at 2 and 6-weeks to baseline, a significant shift in the community profile was observed for zinc toothpaste users (*p* = 0.002 and *p* = 0.001, respectively) but no shift for control toothpaste users (*p* = 0.899 and *p* = 0.936). No difference was observed for either toothpaste between 2 and 6-weeks (*p* = 0.973 for the zinc group and *p* = 1.000 for the control group).

To understand the community differences observed, the mean relative abundance (MRA) of individual species was compared between toothpastes over time. The 584 species-level taxa were examined for changes in mean relative abundance from baseline to 2- and 6-weeks, for each toothpaste, using Dirichlet Multinomial algorithm^24^. The positive false discovery rate was tightly controlled to within 5% using the q-value adjusted p-value method^25^. A filter was applied to the significant taxa identified to assess only the most relevant (prevalence > 30% and abundance > 0.005%). Significant changes in MRA were observed for 38 taxa, 30 for the zinc toothpaste and 8 for control toothpaste (Supplementary Table S1 and S2). Two species were identified as having changed in MRA for both toothpastes, namely *Staphylococcus epidermidis* and *Haemophilus haemolyticus.*

For each toothpaste, significant taxa were assessed in terms of increase or decrease in MRA compared to baseline. At 2-weeks, 13 taxa increased and 8 decreased for the zinc toothpaste, while only 1 species increased and 1 decreased for the control toothpaste. At 6-weeks, 16 taxa increased and 8 decreased for the zinc toothpaste, while 1 taxon increased and 5 taxa decreased for the control toothpaste, although all changes were under 0.05% in this latter group. All changes are captured in Supplementary Tables S1 and S2.

**Fig. 2:**
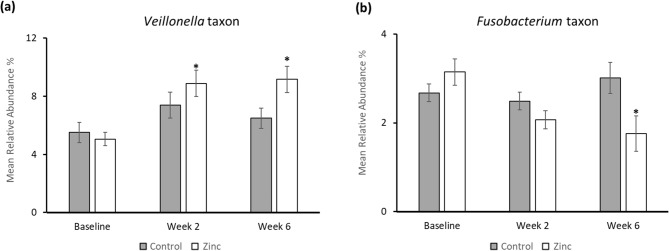
Mean relative abundance (% MRA) mean (standard error) at species level for the most significant increased and decreased taxa with the use of the control (grey bars) and zinc toothpaste (white bars) over time (a) *Veillonella taxon (Veillonella_dispar|parvula|sp._Oral_Taxon_E53 |sp._str._3144|sp._str._6127)*, (b) *Fusobacterium nucleatum* subsp. *polymorphum*. Significant differences compared to baseline (*p* < 0.05) are highlighted *.

The MRA of 3 taxa increased by more than 1% for the zinc toothpaste at both 2 and 6-weeks compared to baseline with the largest increase being a taxon from the genus *Veillonella* with a 3.8% and 4.1% change, respectively (Fig. 2a). Two other *Veillonella* taxa also increased in MRA for the zinc toothpaste; no significant increases were observed in *Veillonella* taxa for control toothpaste. Taxa from other genera including *Campylobacter* and streptococci were also increased in MRA, but to a lesser extent following use of the zinc toothpaste.

Two species were reduced by over 1% in MRA following the use of the zinc toothpaste with the largest reduction being *Fusobacterium nucleatum* subsp. *polymorphum* after 6 weeks with a decrease of 1.4% (Fig. 2b). The second species was *Neisseria elongata*, which was only significantly reduced at the 2-week time point. Two taxa from the genus *Porphyromonas* were found to decrease in MRA for the zinc toothpaste, *P. pasteri* at week 6 and *Porphyromonas* sp. oral taxon 275 at both 2 and 6-weeks. For the control toothpaste, five taxa decreased in MRA, but all changes were under 0.05% (Supplementary Table S2).

### Change in predicted metagenomes

**Fig. 3:**
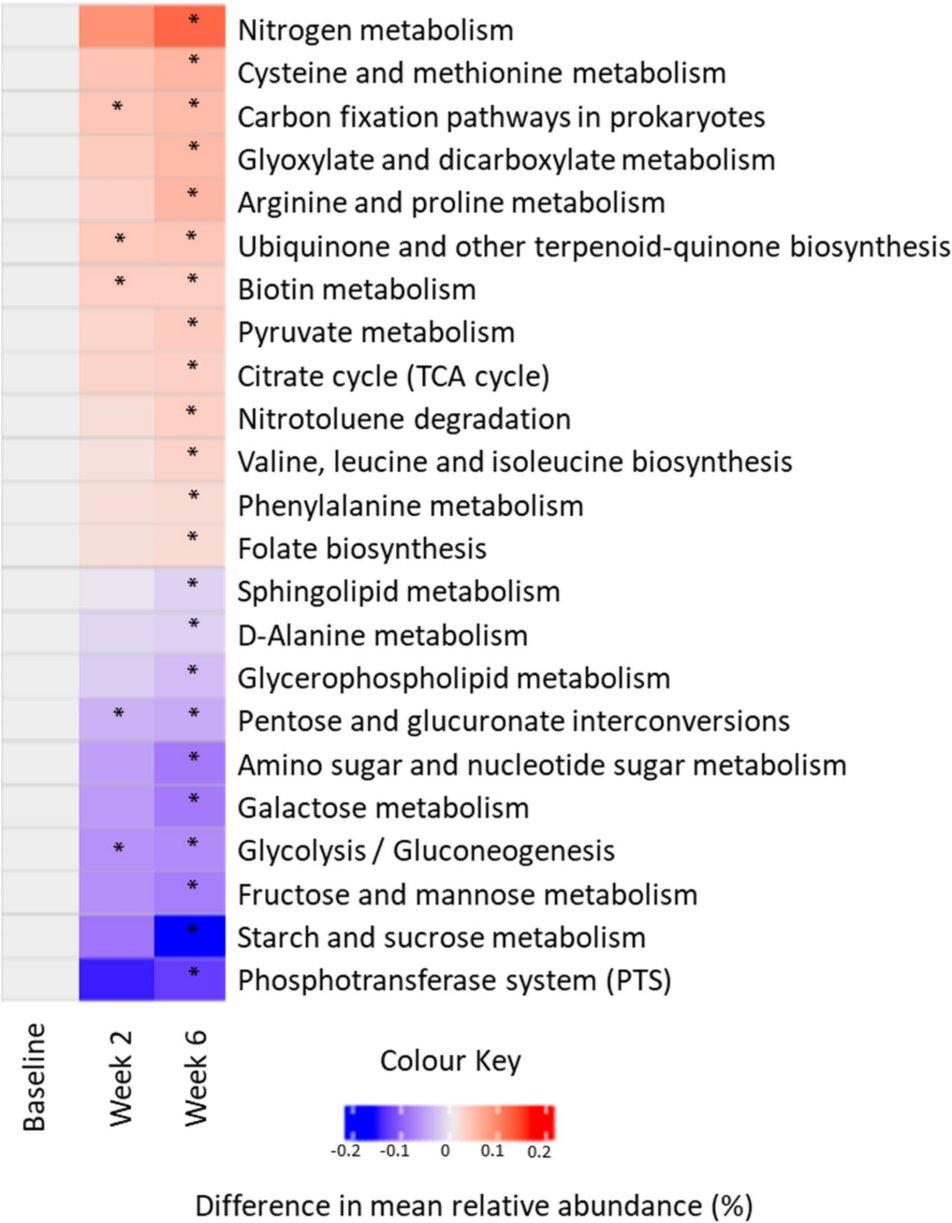
Differences in mean relative abundance (%) of selected KEGG pathways between the zinc and control toothpaste at each of the baseline, week 2 and week 6 timepoints. Pathways highlighted (*) show statistically significant differences in LEfSe analysis.

Tax4Fun was used to predict the metagenome of the clinical samples based on metataxonomic profile^13^. Two hundred and seventy KEGG pathways were identified from the data and were analysed in paired sets using LEfSe to determine significant differences. From the output generated by LEfSe for the baseline samples, only 2 pathways, one per product, were significantly different. However, with toothpaste use, increasing numbers of pathways were found to be significantly different between toothpastes. At 2 weeks, 6 pathways were significantly different between products, 4 increased for the zinc toothpaste and 2 increased for control users; at 6 weeks, 26 pathways were significantly different between products, 15 increased for the zinc toothpaste and 11 increased for those in the control group (Supplementary Table S3 and Fig. 3). The changes included glycolysis (map00010), fructose and mannose metabolism (map00051), starch and sucrose metabolism (map00500), amino sugar and nucleotide sugar metabolism (map00520) and phosphotransferase system (PTS) (map02060) which were decreased following use of the zinc toothpaste compared to control, a trend consistent with the mode of action of zinc^26^. Interestingly, some pathways not usually linked to the mode of action of zinc were observed to increase for the zinc product including nitrogen metabolism (map00910) and pathways associated with amino acid and vitamin metabolism.

Comparing toothpastes over time, a greater number of differences were observed for zinc toothpaste users compared to baseline. This is in line with expectations based on the number of changes in mean relative abundance of taxa for the zinc compared to the control toothpaste. At the 2-week time point, compared to baseline, there were 43 pathways changed for the zinc toothpaste (24 increased and 19 decreased compared to baseline) and 6 for control (all decreased compared to baseline). At the 6-week time point this increased to 50 pathways for the zinc toothpaste (23 increased and 27 decreased compared to baseline) and 8 for control (4 increased and 4 decreased) (Supplementary Table S4). The pathways increased for the zinc toothpaste compared to baseline at both time points included nitrogen metabolism (map00910) and lysine biosynthesis (map00300). Furthermore, lysine degradation (map00310) and pathways linked to sugar metabolism including glycolysis (map00010), starch and sucrose metabolism (map00500) and the PTS system (map02060) decreased after 2- and 6-weeks use of zinc toothpaste.

### Metatranscriptomic analysis

The study of functional output of bacteria using metagenomics and metatranscriptomics allows understanding in terms of both what the community is capable of and what the community is doing at that time point. Statistical analysis was carried out on all genes present using DESEQ2 to identify pathways with significant differences between zinc and control toothpastes. A total of 463 KEGG pathways were identified from the data set and were analysed in paired sets. From the output generated by DESEQ2, when comparing between toothpastes at baseline, 2 pathways were significantly upregulated in the zinc toothpaste samples and 11 significantly downregulated relative to control. Following toothpaste use, increasing numbers of pathways were found to be significantly different between toothpastes, with 36 pathways significantly upregulated and 30 significantly downregulated following use of the zinc toothpaste compared to control. At 6 weeks, 22 pathways were upregulated, and 12 pathways were downregulated in the zinc toothpaste samples compared to control samples. These included a significant reduction in glycolysis and lysine degradation, and a significant increase in nitrogen metabolism.

### Combination of predicted metagenome (Tax4Fun) and metatranscriptome

**Fig. 4:**
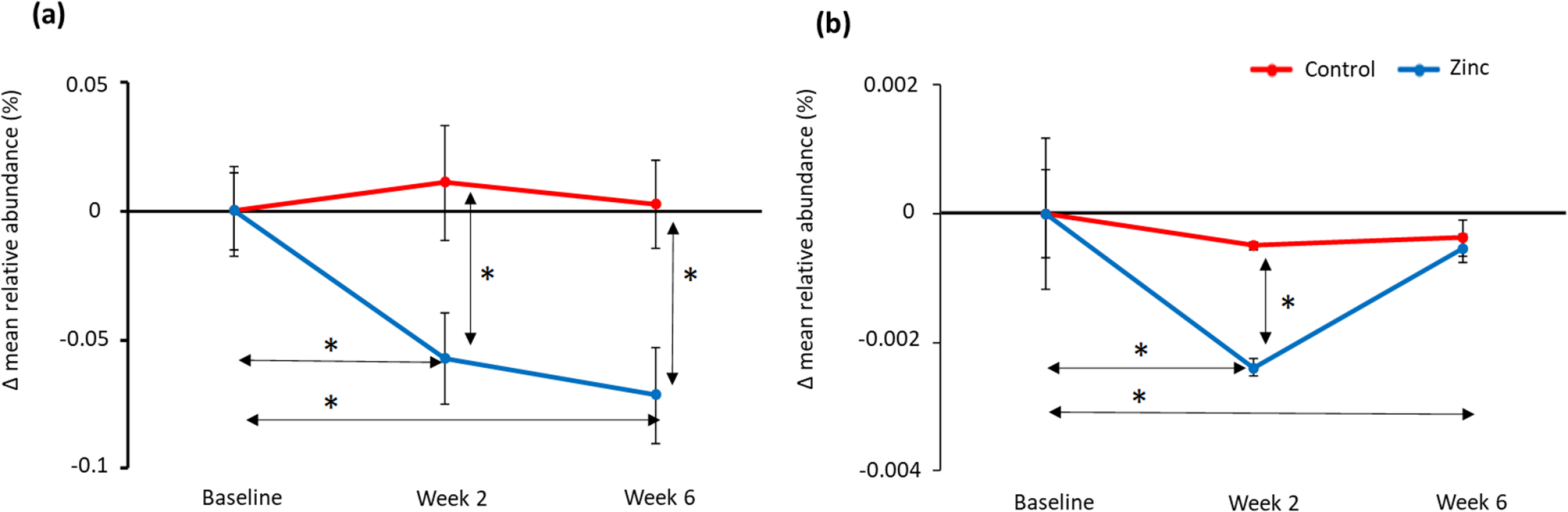
Changes in mean relative abundance (%) levels of glycolysis pathway genes for the control toothpaste (red) and zinc toothpaste (blue) compared to baseline, based on analysis using Tax4Fun (a) and metatranscriptomics (b). Significant differences (*p* < 0.05) are highlighted * and error bars represent standard error.

Changes in the glycolysis/gluconeogenesis pathway (map00010) were observed by both Tax4Fun and the metatranscriptome analysis with significant differences between toothpastes and over time (Fig. 4). Using Tax4Fun, no difference was observed at baseline between toothpastes, but significant differences were observed between toothpastes at both subsequent time points, as well as significant reduction for both 2- and 6-weeks compared to baseline. A similar picture was observed for the metatranscriptome analysis over time and between toothpastes, which is consistent with the mode of action of zinc.

**Fig. 5:**
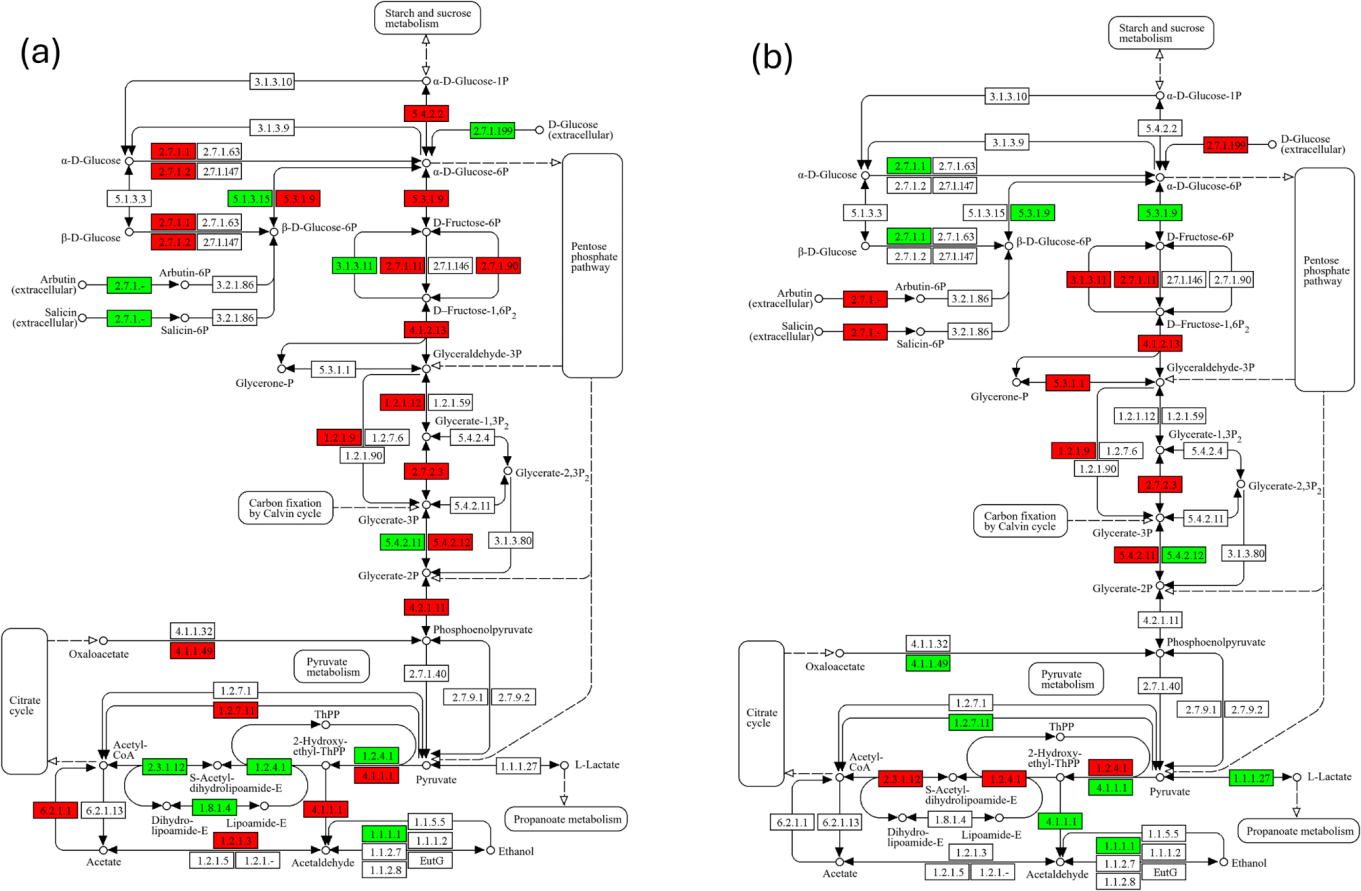
Differential expression of metatranscriptomic genes found in the glycolysis pathway using KEGG mapper^64^. Green gene labels show increases in expression, with red gene labels showing a decrease in expression; white gene labels indicate no significant change between zinc toothpaste group and control group at (a) 2 weeks and (b) 6 weeks.

Using metatranscriptomic data it is possible to explore individual genes present in a pathway and changes over time and with toothpaste use can then be identified. Figure 5 illustrates such an analysis using the glycolysis/gluconeogenesis pathway (map00010) highlighting genes affected using the zinc toothpaste compared to control with increased expression highlighted in green and decreased in red.

As well as significantly inhibiting the glycolytic activity of the dental biofilm compared to control, the zinc toothpaste also led to significant downregulation of other sugar pathways including fructose metabolism (map00051), galactose metabolism (map00052), amino sugar and nucleotide sugar metabolism (map 000520), the phosphotransferase system (PTS) (map 02060) and starch and sucrose metabolism (map 00500). The opposite trend was observed for the citrate TCA cycle and pyruvate metabolism pathways where a significant upregulation was observed for the zinc product compared to control and baseline.

**Fig. 6:**
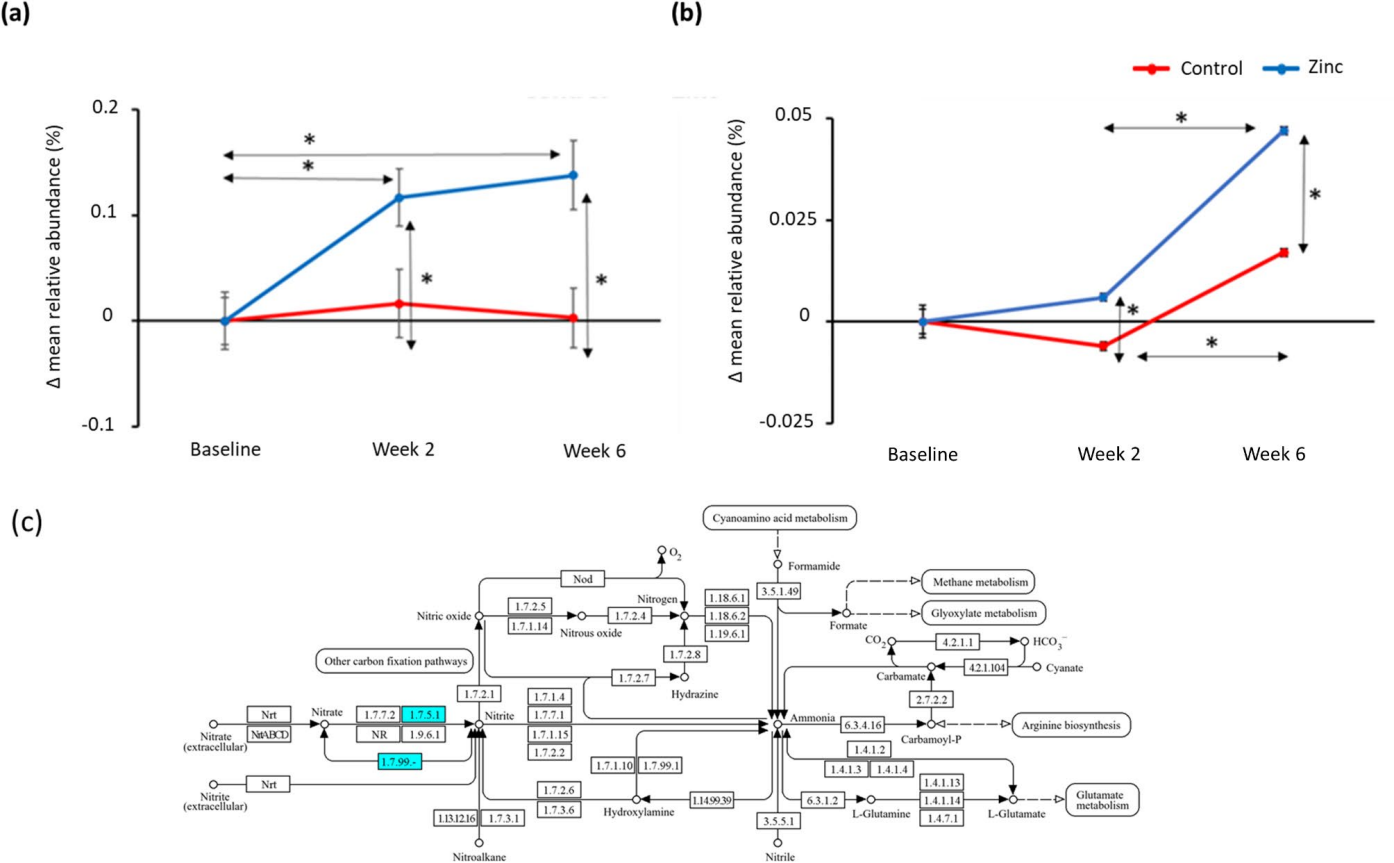
(a) Changes in mean relative abundance (%) levels for the nitrogen metabolism pathway for the control toothpaste (red) and zinc toothpaste (blue), based on analysis using Tax4Fun and (b) Metatranscriptome changes MRA (%) of the nitrate reductase gene (E.C: 1.7.5.1) for the control toothpaste (red) and zinc toothpaste (blue). Significant differences (*p* < 0.05) are highlighted * and error bars represent standard error. (c) Displays the nitrogen metabolism KEGG map with the nitrate reductase gene highlighted in blue.

Nitrogen metabolism (map00910) changes were identified using LEfSe from the Tax4Fun data and plotted to understand the changes over time and by toothpaste. Figure 6a describes the changes in nitrogen metabolism, with a significant increase for the zinc toothpaste over time and compared to the control toothpaste. The nitrogen metabolism pathway (map00910) has many steps but of particular relevance is the conversion of nitrate to nitrite. Again, using metatranscriptomic data we can examine the changes in expression during the study for the individual gene encoding this conversion (E.C. 1.7.5.1). Figure 6b, illustrates the significant up-regulation of nitrate reductase gene both over time and between toothpastes at both time points.

## Discussion

Previously, Adams et al.^11^ demonstrated a significant shift in ecology of the oral microbiome following use of a toothpaste formulation containing enzymes and proteins. The work presented here builds on metataxonomic techniques reported for the investigation of the impact of toothpastes on the oral microbiota by adding metagenome prediction and metatranscriptome analysis. These techniques were used to examine the effect of a toothpaste formulation containing 2% zinc citrate trihydrate on dental biofilm composition and activity compared to a control toothpaste. This is, to our knowledge, the first study to combine the use of metataxonomics and metatranscriptomics to identify changes in microbiome gene expression in a human clinical trial both over time and when comparing a zinc toothpaste to a control toothpaste.

A recent study by Mira et al.^16^ examined the effect of toothpaste use on microbial function of human dental plaque. As part of their observations, they discussed the length of washout periods before collection of samples for microbiome analysis. Their study included a 1-week washout with fluoride toothpaste before collection of baseline plaque samples followed by 3 months on the same product and collection of further samples. They observed changes in the microbiome between these sampling points and questioned whether a 1-week washout is sufficient to rule out changes from the control product. In this study subjects undertook a 4-week washout prior to collection of samples for microbiome analysis; no difference in community profile was observed with minimal differences in taxa over time for the control product. Therefore, we concur that a longer washout is desirable and believe that a 4-week washout period provides a stable microbiome for comparison with more active toothpaste formulations, ruling out the effect of toothbrushing as a confounding factor^11,16^.

Following the use of zinc toothpaste, changes in the microbiome were observed compared to control toothpaste. Shifts in community profiles were found for the zinc toothpaste both over time and in comparison to control toothpaste with no change observed for the control toothpaste; this mirrors the earlier study of Adams et al.^11^. Significant changes in mean relative abundance of 30 taxa were observed following the use of zinc toothpaste with only 8 significant changes for control toothpaste; this is consistent with the stable community composition for the control toothpaste. Alongside community and species level changes, analysis at the functional level allows examination of product effects previously associated with the mode of action of zinc salts, such as reduction in dental biofilm acid production and new insights around oral and whole-body health.

The change to taxonomic composition of the microbiome, as it regrows following twice daily brushing with a zinc toothpaste may be predicted based on the wealth of literature for zinc salts as bacteriostatic agents^26,27^. Although the changes are small, they are statistically significant. Our data suggest that zinc, unlike other bactericidal agents, has a selective mode of action, restricting growth of some species, making them less competitive, without killing them, and therefore giving other species a selective growth advantage. The change in community appears to take place over the initial 2-week period and stabilises between 2 and 6-weeks of toothpaste use, with changes compared to baseline for both time points but no change between 2 and 6-weeks. The observed change in community composition indicates that the use of a zinc toothpaste has a small but significant impact on relative abundance of individual species within the ecosystem.

Interestingly, for the majority of bacterial species which significantly increase in MRA with use of the zinc toothpaste, the same pattern was observed where, following the initial increase between baseline and week 2, the species stabilised at the new higher level between weeks 2 and 6. This pattern of increase and stabilisation is exemplified by Fig. 2 for the *Veillonella* taxa (also in Supplementary Table S1) and suggests that changes observed in the zinc group represent a sustainable shift in overall ecology of the plaque biofilm.

The increase in relative abundance of *Veillonella* species in dental plaque following use of a zinc toothpaste has been observed before, at the genus level, using standard microbiological techniques^10^. Exploring the genome of oral *Veillonella*, *V. parvula*,* V. dispar* and *V. atypica*, reveals that all possess two zinc transport systems, ZnuABC (zinc only) and TroACDB (zinc, manganese and iron), allowing regulation of the levels of zinc in the cell and survival in the presence of elevated zinc levels^28^. Examination of the response of multiple genes found in these transport systems, using metatranscriptome data, revealed down-regulation of all ZnuABC genes and mixed responses for the TroACDB following use of the zinc toothpaste compared to control toothpaste. Other species are known to possess these transport systems so this may not be the only explanation for the ability of *Veillonella* species to thrive in presence of zinc salts. Greater understanding might come from examining changes in the transcriptome of *Veillonella* species following growth in the presence of zinc salts and observing changes in gene expression. Whilst the overall role of *Veillonella* species has been subject to scientific discussion^29–32^ it is recognised as a core genus associated with a healthy mouth^9,33–35^. Therefore, the increase in proportion of *Veillonella* taxa observed in this study can be seen as beneficial and potentially an additional mechanism contributing to overall efficacy of zinc toothpaste as part of an effective oral health regimen. Species from other oral genera were also found to increase, but to a lesser extent following the use of the zinc toothpaste, including *Campylobacter gracilis*. This species has been considered as potentially either a member of the orange complex or as core member of the oral microbiome, abundant in both health and disease^14,36,37^. The lack of consensus may in part by due to differences in spatial localisation and health associations between supragingival and subgingival plaque and highlights the need for further investigation to robustly define the health association of this organism.

The species found to reduce most significantly following the use of a zinc toothpaste was *Fusobacterium nucleatum* subsp. *polymorphum* (Fig. 2). Whilst this is a core oral microbe, it is linked to maturation of the developing biofilm to contain more obligately anaerobic species, many of which are associated with inflammation and gum disease^38^. The reduction in levels of *Fusobacterium* species suggests that the biofilm formed following use of the zinc toothpaste may contain fewer obligate anaerobes and higher proportions of species associated with a healthy oral microbiome. *Fusobacterium nucleatum* ATCC 25586 has previously been shown to be more sensitive to zinc salts than eight other oral bacteria^39^. Although it is a different subspecies, this observation may provide an insight into why *Fusobacterium* species were affected most following use of a zinc toothpaste. The second most abundant species to decrease with use of the zinc toothpaste was *Neisseria elongata* a species associated with nitrate reduction. This change was only significant at the 2-week timepoint compared to baseline. Two taxa from the genus *Porphyromonas* were also found to decrease in MRA with the zinc product. *P. pasteri* (Oral Taxon 279) has been linked to oral malodour^40^ and was significantly reduced at the 6-week time point compared to baseline.

Several streptococcal taxa showed significant changes in MRA with use of the zinc toothpaste. For example, the *S. dentisani/mitis* taxon which is associated with oral health significantly decreased in MRA after 2 weeks of product use, although there was no difference at 6 weeks; *S. salivarius/vestibularis are* also associated with oral health and significantly increased in MRA 2 weeks of product use, with no difference at 6 weeks.

The findings described here provide the first detailed mechanistic information on the positive contribution of *Veillonella* to oral health. Furthermore, these results highlight potential new insights into how zinc, which has been used in oral care products for decades, exerts a beneficial effect on the oral microbiome by favouring *Veillonella* colonisation to the detriment of *Fusobacterium*^33,41^.

Understanding the link between compositional changes and functional variability in the oral microbiome is of great importance. In the oral cavity many species possess similar functional capabilities, for example, the conversion of glucose to lactate, whilst other species may possess unique functional capabilities. Understanding the taxa present in the microbiome is important to determine shifts between disease states and a healthy microbiome. However, recognising the effect of treatment on orally relevant endpoints, such as acid production linked to caries, at community or “meta” level, is of great value. The use of active agents like zinc salts, which are bacteriostatic and are known to inhibit enzymes, enables beneficial changes in the functional profile of communities.

The functional output of the bacterial communities in this study was followed using prediction of the metagenomes via Tax4Fun and metatranscriptomic analysis. With both methods, inhibition of glycolysis was observed after using the zinc toothpaste both over time and compared to control toothpaste (Fig. 4). From metatranscriptomic data, changes in individual genes present in the glycolytic pathway were tracked and differences between the toothpastes after both 2- and 6-weeks assessed. By the 6-week time point, all bar two genes of the glycolytic pathway were downregulated compared to control (Fig. 5). The final gene in the pathway, L-lactate dehydrogenase E.C.1.1.1.27, which catalyses the conversion between pyruvate and lactate, is up-regulated in the presence of zinc salts. This is a reversible reaction and with the increase in levels of *Veillonella* species in the community, increased metabolism of lactate may be expected as this is the favoured carbon source of this genus^42^. The metabolism of lactate by *Veillonella* leads to production of weaker acids like propionic and acetic, that are less damaging to enamel than lactic acid^42,43^. The impact of zinc on glycolysis is a well-documented mode of action with clinical studies observing a reduction in acid production by plaque following rinsing with zinc and a subsequent carbohydrate challenge^44,45^. However, not so well understood is the effect of zinc on other sugar metabolism and transport pathways. From analysis using Tax4Fun, several other sugar metabolism and transport pathways were reduced for zinc toothpaste compared to control toothpaste and to baseline values; these included fructose and mannose metabolism (map00051), starch and sucrose metabolism (map00500), amino sugar and nucleotide sugar metabolism (map00520) and the phosphotransferase system (PTS) (map02060) (Supplementary Tables S3 and S4). Although these pathways do not generate acid directly, they can provide metabolites for use within the glycolysis pathway. Therefore, it is possible that inhibiting the wider sugar metabolism network may lead to even less acid production than glycolysis inhibition alone. Interestingly, the citrate (TCA) cycle is affected in the opposite direction with a significant increase following use of zinc toothpaste both compared to baseline and to control with no change over time for control toothpaste. As the TCA cycle is a strictly aerobic process this observation may imply a less mature and more aerobic plaque formed with the use of a zinc toothpaste, which is consistent with the reduction in *Fusobacterium*. Although *Veillonella parvula* is anaerobic, it is predicted to contain several enzymes which form part of the TCA pathway including pyruvate carboxylase which converts pyruvate to oxaloacetate and is the starting point of the TCA cycle. On examining the pyruvate metabolism pathway and the TCA cycle, it was observed that both cycles increase over time with the zinc toothpaste but remain stable with the control toothpaste. The cysteine and methionine metabolism (map00270) pathway was also found to significantly increase following use of zinc toothpaste both compared to baseline and to control with no change over time for the control toothpaste. This is a complex pathway with many processes; however, zinc is known to bind sulphur compounds and the overall increase in the pathway maybe a response to this mode of action.

Other functional processes can be beneficial to the oral cavity, for example lysine biosynthesis, which has been linked to gum health; similarly, lysine degradation has been linked to impaired gum attachment^46^. Lysine is essential for growth of human cells, but they are incapable of synthesising lysine de-novo, instead deriving lysine in health from interstitial fluid and from gingival crevicular fluid during inflammation. Tax4Fun analysis of the current study data shows an increase in lysine biosynthesis and decrease in lysine degradation, which was confirmed by metatranscriptome analysis for subjects using the zinc toothpaste compared to control toothpaste and baseline. Lohinai et al.^46^ observed that upon restriction of oral hygiene, there is a decrease in lysine concentration and increase in cadaverine, linked in turn to an increase in lysine decarboxylase activity and biofilm accumulation. Interestingly, there appears to be a positive relationship between dental biofilm lysine concentration, minimal blood plasma levels, epithelial barrier integrity and quantity of gingival crevicular fluid. When biofilm lysine is greater than minimal blood plasma levels the gum barrier remains intact and less GCF is produced^46^. Hence, the increase in the ability of plaque biofilm to produce lysine may provide a route to maintaining healthier gums.

As well as pathways beneficial to a healthy oral cavity, the oral microbiome plays a part in whole body health. One such area is the conversion of nitrate to nitric oxide which has been studied since the 1970’s^47^. Dietary nitrate is absorbed into the blood and secreted by the salivary glands from plasma; oral bacteria convert the nitrate into nitrite and further into nitric oxide which is known to have influence on a number of biological processes, including blood pressure and diabetes^48,49^. A wide range of studies on the oral microbiome and its ability to reduce nitrate to nitrite have been conducted, examining changes in diet^50^, blood pressure^51^ and oral hygiene products^52^ amongst others. Studies have examined the use of nitrate as a prebiotic and nitrate-producing bacteria as potential probiotics^53,54^. From the literature, *Veillonella* is one of the most common nitrite-producing oral genera, along with *Neisseria* and *Rothia* and an increase in these bacteria should provide an increase in nitrate reduction. A study by Vanhatalo examined the effect of nitrate supplementation of the diet on the oral microbiome and found increases in relative abundance of *Neisseria* and *Rothia* species but interestingly a decrease in *Veillonella*^55^. From the study reported here, we observed changes in nitrogen metabolism and specifically an upregulation in nitrate reductase gene following use of zinc toothpaste for 6-weeks alongside an increase in *Veillonella* species, without need for additional nitrate supplementation. This implies a further beneficial mode of action for toothpaste containing 2% zinc citrate trihydrate. Future investigation into this mode of action is needed to confirm that increased conversion of nitrate to nitrite in vivo is observed following use of a toothpaste containing 2% zinc citrate trihydrate.

One possible limitation of this study is the lack of a corresponding clinical measure from the same cohort, for example plaque pH, to confirm the observation of reduced glycolysis in those subjects using the zinc product. However, there is a wealth of existing data on the ability of zinc to inhibit acid production^22,26,44,45,56^. Studies examining toothpaste containing zinc citrate have confirmed the ability of zinc to inhibit the pH response of plaque^17^ and to inhibit plaque lactic acid production compared to a control following consumption of a food-derived carbohydrate^20,57^. Additional studies have examined the delivery of zinc to plaque following food consumption and the zinc levels delivered were shown in vitro to inhibit the ability of salivary bacteria to generate acid^58,59^. Additionally, toothpastes containing zinc citrate alone have been found to inhibit plaque formation and to have a beneficial effect on gum heath with studies using 0.5% zinc citrate trihydrate giving up to 22 h of plaque reduction compared to a control toothpaste without zinc^5^. More recently, Schafer et al.^60^ studied the effect of 2% zinc citrate on plaque levels and gum health. These studies found (a) a reduction in plaque compared to a control toothpaste and (b) after 3-months the zinc citrate trihydrate containing toothpaste reduced plaque and gingival inflammation relative to baseline and was as effective as a 0.3% Triclosan toothpaste. The combination of microbiome and clinical measures in a future study would strengthen the conclusions possible from the study.

## Conclusion

The work presented here provides new insights into both the microbial and functional changes in dental biofilms following use of a zinc toothpaste and into mechanisms by which oral hygiene products containing zinc salts may exert beneficial effects on oral health. Specifically, significant increases were observed in three *Veillonella* taxa, with their ability to reduce the levels of harmful plaque acids, alongside significant reduction in *Fusobacterium nucleatum* subsp. *polymorphum* with the ability to bridge to more harmful species linked to gum disease. The work further demonstrates changes in the bacterial community transcriptome, providing understanding of the functional output of the whole plaque microbiome. These observations reveal: first, inhibition by a zinc toothpaste of glycolysis at the gene level, a well-known mode of action, now demonstrated in vivo, and second, for the first time, the ability of a zinc toothpaste to boost genes associated with the biosynthesis of lysine, which is linked to gum attachment, and of nitrate reductase which has benefits for whole body health. The study of the plaque microbiome at the pathway and gene level has generated new insights into the role of zinc salts in a toothpaste with benefits for not just the oral cavity but potentially for whole-body health.

## Methods

### Ethics statement

Written informed consent was obtained from all enrolled individuals. The study protocol was reviewed and approved by the Manchester Consumer Healthcare Research Ethics Committee and performed according to the principles of both the Declaration of Helsinki and Good Clinical Practice. All relevant guidelines and regulations were followed. Trial registration number NCT06358742 (11/04/2024).

### Participants

Subjects in good health aged 18–65 were recruited onto the study. No formal sample size calculation was performed as suitable data were not available.

Key inclusion criteria included: aged between 18 and 65 years, minimum number of teeth 20, no antibiotic therapy or food supplements containing zinc within 4 weeks of the start of the study. Key exclusion criteria included: pregnancy, nursing mothers, diabetics, denture wearers, smoking within the last 12 months, medical conditions and/or regular use of any medication which might affect the outcome of the study and obvious signs of untreated caries/ significant periodontal disease and subjects with a modified gingival index score (MGI) score of 3 or more at screening^61^.

### Study design

This study was a double-blind (subject, clinician collecting plaque, sample analyst), randomised, parallel group study conducted by an independent UK clinical research organisation from January to May 2017. After recruitment, subjects (*n* = 142) were given a fluoride toothpaste without gum care agents to use for the 4-week run-in period prior to the commencement of the test phase to ensure that all subjects were standardised to a fluoride-only toothpaste. Following the run-in period, baseline supragingival plaque samples were collected 12 h after last brushing, from the upper and lower jaws separately, for metataxonomic and metatranscriptomic analysis, respectively. Samples were collected using a standard dental instrument; the upper jaw samples were placed in 1 ml Tris-EDTA buffer pH7.4 buffer in a 7 ml bijou bottle and stored at – 25 °C until analysis. For the lower jaw samples, each was placed into 0.5 ml Qiagen RNA bacterial protect solution in a low DNA binding Eppendorf and stored at – 25 °C until analysis. Subjects were randomly allocated by CRO staff not involved in sample collection and analysis, to one of two products according to a randomization table generated by study statistician using Proc Plan procedure in SAS 9.4 (SAS Institute Inc., Cary, NC, USA) suitable for 2-group parallel design study. The sequence of product codes was not concealed, however individuals allocating product to subjects were not involved in sample analysis. The two products were a fluoride toothpaste (1450 ppm) containing 2% zinc citrate trihydrate (zinc toothpaste) or an otherwise identical control fluoride toothpaste (1450 ppm) but without zinc. Both toothpastes were white products provided in labelled plain white laminate tubes which only differed in the code on the label. Subjects were instructed to use the toothpaste at home, brushing twice a day for 6-weeks. Supragingival plaque samples were collected again from the upper and lower jaws after 2 and 6 weeks.

### Metataxonomic analysis

DNA was extracted from the plaque collected from the upper jaw as detailed in Adams et al.^11^. Following extraction, the bacterial DNA levels were quantified using Quantitative Polymerase Chain Reaction (qPCR) using primers targeting the V1-V2 region of the 16S rRNA gene. Oligonucleotide primers targeting the V1–V2 hypervariable region of the 16S rRNA gene were used to generate amplicons for sequencing. Positive (in-house mock community) and negative controls were included in all PCR batches. Production of libraries for sequencing was as detailed in Adams et al.^11^ with each pool of libraries sequenced on one flowcell of an Illumina MiSeq with 2 × 250 bp paired-end sequencing using v3 chemistry (Illumina, California, USA).

Raw sequencing reads were processed simultaneously on a bespoke pipeline hosted by Eagle Genomics, outlined in the Supplementary Information. Statistical analyses were performed on the table of counts produced from the bioinformatics pipeline using methods described in Adams et al.^11^. Counts were analysed at the species level. Community changes were assessed using analysis of variance (ANOVA) to determine the statistical significance using a between sample distance measure (Bray Distance) based on taxonomic profile. Model-based approaches were employed for comparative testing of relative abundance between groups which explicitly model the uneven sample read numbers and sparseness. KEGG pathway analysis based on the Tax4Fun output was analysed using linear discriminant analysis effect size (LEfSe) to identify biomarkers between the sample groups^62^.

### Metatranscriptomic analysis

Plaque from the lower jaw of the 115 subjects completing the study were pooled to generate 6 samples: baseline, 2 and 6 weeks for each toothpaste. Each pool was split into 3 aliquots and RNA extracted using the Qiagen AllPrep kit following the manufacturer’s guidelines (Qiagen, Germany). Quantification was performed using a Quant-IT high sensitivity RNA Assay kit (Invitrogen, California, USA) following manufacturer’s guidelines. Libraries were generated using NEBNext Ultra Directional RNA library prep kit for Illumina protocol with rRNA deletion using the Epidemiology kit. Libraries were sequenced on two lanes of an Illumina HiSeq 4000 with 2 × 150 bp paired-end sequencing (Illumina, California, USA).

Metatranscriptomic reads from all 18 samples were processed simultaneously on a bespoke pipeline, hosted by Eagle Genomics, outlined in the Supplementary Information. Differential gene expression analysis was performed using the Bioconductor package DESEQ2 version 1.26.0^63^.

## Electronic supplementary material

Below is the link to the electronic supplementary material.

Supplementary Material 1

## Data Availability

The datasets generated during the current study are available in the SRA repository, submitted under BioProject: PRJNA1024507.
