# Supplementary material for: A randomised, double-blind clinical study into the effect of zinc citrate trihydrate toothpaste on oral plaque microbiome ecology and function
Source: Sci Rep. 2025 Mar 8;15:8136. doi: 10.1038/s41598-025-92545-0 (PMC11890769; doi:10.1038/s41598-025-92545-0)
Supplement: Supplementary file 1 — Supplementary Material 1 [file 41598_2025_92545_MOESM1_ESM.pdf]

A randomised, double-blind clinical study into the effect of zinc citrate trihydrate toothpaste on oral plaque microbiome ecology and function

Adams, SE[1], Cawley, AK[1], Arnold, D[1], Hoptroff, MJ[1], Slomka, V[1], Matheson, JR[1], Marriott, RE[1], Gemmell, MR[2], Marsh, PD[3]

[1] Unilever Oral Care, Bromborough Road, Bebington, Wirral. CH63 3JW. UK.

[2] Institute of Integrative Biology, Centre for Genomic Research, University of Liverpool, Crown Street, Liverpool L69 7ZB, UK

[3] Department of Oral Biology, School of Dentistry, University of Leeds, Leeds, LS2 7TF, UK

Supplementary Material

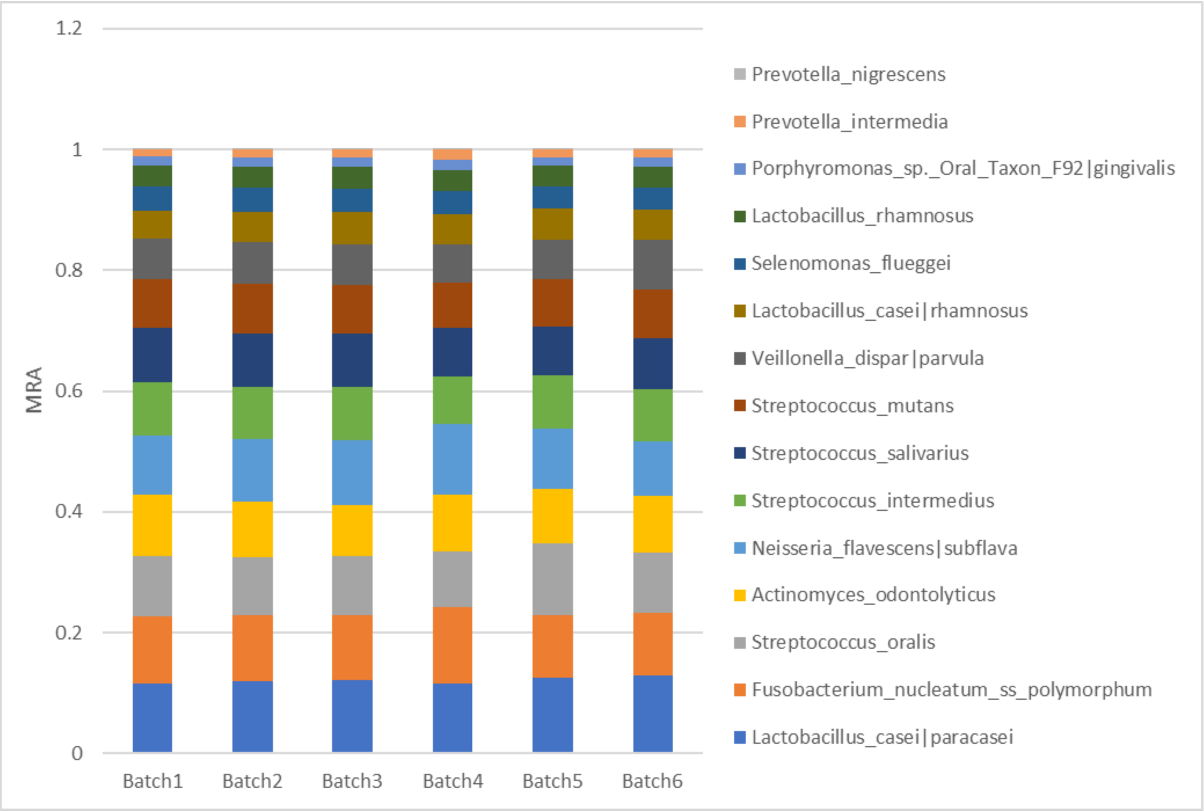

Figure S1 - Batch to batch comparison of the positive control mock communities, based on the mean relative abundance (MRA) for the 15 species present. The mock community was present in all the 6 sequencing runs, with excellent consistency between batches.

Table S1 - Changes in mean relative abundance observed for the significantly different taxa following use of the zinc toothpaste at 2 and 6 weeks compared to baseline ($p<0.05$ and $q<0.05$).

<table>
<tr><th>Taxa</th><th>Change in mean relative abundance baseline - 2 weeks (%)</th><th>Mean relative abundance at baseline (%)</th><th>Mean relative abundance after 2 weeks (%)</th><th>p values</th><th>q values</th><th>Change in mean relative abundance baseline - 6 weeks (%)</th><th>Mean relative abundance after 6 weeks (%)</th><th>p values</th><th>q values</th></tr>
<tr><td>Veillonella_dispar|parvula|sp._Oral_Taxon_E53|sp._str._3144|sp._str._6127_OTU_73</td><td>3.830</td><td>5.057</td><td>8.887</td><td>0.000294</td><td>0.00692</td><td>4.104</td><td>9.160</td><td>0.000246</td><td>0.003459</td></tr>
<tr><td>Actinomyces_sp._oral_taxon_448_OTU_29</td><td>1.185</td><td>0.775</td><td>1.959</td><td>0.018086</td><td>0.137686</td><td>1.789</td><td>2.564</td><td>8.35E-05</td><td>0.001485</td></tr>
<tr><td>Veillonella_parvula_OTU_94</td><td>1.424</td><td>1.100</td><td>2.524</td><td>3.43E-05</td><td>0.001159</td><td>1.190</td><td>2.290</td><td>0.000307</td><td>0.004152</td></tr>
<tr><td>Campylobacter_gracilis|sp._Oral_Taxon_E67_OTU_39</td><td>0.665</td><td>0.879</td><td>1.545</td><td>0.000511</td><td>0.009527</td><td>0.764</td><td>1.643</td><td>0.001327</td><td>0.015057</td></tr>
<tr><td>Streptococcus_tigurinus_OTU_315</td><td>0.385</td><td>0.264</td><td>0.649</td><td>0.025578</td><td>0.17725</td><td>0.632</td><td>0.896</td><td>0.000311</td><td>0.004152</td></tr>
<tr><td>Prevotella_histicola_OTU_314</td><td>0.119</td><td>0.070</td><td>0.188</td><td>0.209696</td><td>0.563032</td><td>0.591</td><td>0.661</td><td>0.00163</td><td>0.017383</td></tr>
<tr><td>Campylobacter_gracilis_OTU_132</td><td>0.257</td><td>0.099</td><td>0.356</td><td>0.00265</td><td>0.03581</td><td>0.407</td><td>0.506</td><td>0.000157</td><td>0.002322</td></tr>
<tr><td>Streptococcus_anginosus_OTU_229</td><td>0.487</td><td>0.064</td><td>0.550</td><td>5.57E-07</td><td>3.01E-05</td><td>0.382</td><td>0.445</td><td>2.91E-08</td><td>1.03E-06</td></tr>
<tr><td>Prevotella_sp._oral_taxon_313_OTU_309</td><td>0.201</td><td>0.010</td><td>0.211</td><td>0.000738</td><td>0.012473</td><td>-0.001</td><td>0.009</td><td>0.955403</td><td>0.923424</td></tr>
<tr><td>Streptococcus_salivarius|vestibularis_OTU_111</td><td>0.197</td><td>0.016</td><td>0.214</td><td>0.000398</td><td>0.007676</td><td>0.085</td><td>0.101</td><td>0.034368</td><td>0.172422</td></tr>
<tr><td>Megasphaera_micronuciformis_OTU_100</td><td>0.103</td><td>0.036</td><td>0.139</td><td>0.001217</td><td>0.018796</td><td>0.158</td><td>0.194</td><td>0.00015</td><td>0.002289</td></tr>
<tr><td>Atopobium_parvulum_OTU_353</td><td>0.049</td><td>0.015</td><td>0.064</td><td>8.71E-05</td><td>0.00277</td><td>0.100</td><td>0.115</td><td>1.87E-07</td><td>6.24E-06</td></tr>
<tr><td>Selenomonas_sp._oral_taxon_134_OTU_78</td><td>0.045</td><td>0.037</td><td>0.082</td><td>0.070068</td><td>0.313341</td><td>0.089</td><td>0.125</td><td>0.001385</td><td>0.015384</td></tr>
<tr><td>Lachnospiraceae_[G-2]_sp._oral_taxon_096_OTU_227</td><td>0.007</td><td>0.011</td><td>0.019</td><td>0.613616</td><td>0.830885</td><td>0.079</td><td>0.090</td><td>0.003203</td><td>0.031053</td></tr>
<tr><td>Parvimonas_sp._oral_taxon_110_OTU_287</td><td>0.080</td><td>0.011</td><td>0.090</td><td>0.003986</td><td>0.046839</td><td>0.023</td><td>0.033</td><td>0.084822</td><td>0.301722</td></tr>
<tr><td>Fusobacterium_naviforme|nucleatum_ss_vincentii_OTU_193</td><td>0.013</td><td>0.001</td><td>0.014</td><td>1.07E-07</td><td>6.4E-06</td><td>0.020</td><td>0.021</td><td>4.12E-13</td><td>3.66E-11</td></tr>
<tr><td>Actinomyces_sp._oral_taxon_181_OTU_512</td><td>0.007</td><td>0.002</td><td>0.009</td><td>0.00362</td><td>0.044467</td><td>0.017</td><td>0.019</td><td>5.23E-07</td><td>1.39E-05</td></tr>
<tr><td>Veillonella_dispar|parvula|sp._str._3144|sp._str._6127_OTU_245</td><td>0.015</td><td>0.019</td><td>0.034</td><td>0.000331</td><td>0.007167</td><td>0.016</td><td>0.036</td><td>0.000196</td><td>0.002832</td></tr>
<tr><td>Streptococcus_sp._Oral_Taxon_71_OTU_178</td><td>0.010</td><td>0.002</td><td>0.012</td><td>0.007353</td><td>0.076435</td><td>0.014</td><td>0.016</td><td>0.001509</td><td>0.01642</td></tr>
<tr><td>Haemophilus_haemolyticus_OTU_269</td><td>-0.024</td><td>0.028</td><td>0.005</td><td>0.000549</td><td>0.009886</td><td>-0.024</td><td>0.004</td><td>0.003947</td><td>0.037585</td></tr>
<tr><td>Haemophilus_haemolyticus|sp._oral_taxon_036_OTU_488</td><td>-0.037</td><td>0.042</td><td>0.005</td><td>1.22E-05</td><td>0.000471</td><td>-0.036</td><td>0.006</td><td>0.005075</td><td>0.047482</td></tr>
<tr><td>TM7_[G-2]_sp._oral_taxon_350_OTU_368</td><td>-0.037</td><td>0.044</td><td>0.007</td><td>0.002811</td><td>0.037061</td><td>-0.041</td><td>0.003</td><td>2.20E-06</td><td>5.34E-05</td></tr>
<tr><td>Porphyromonas_sp._oral_taxon_275_OTU_396</td><td>-0.052</td><td>0.062</td><td>0.010</td><td>0.00012</td><td>0.003603</td><td>-0.056</td><td>0.006</td><td>1.93E-05</td><td>0.00043</td></tr>
<tr><td>Alloprevotella_sp._oral_taxon_473_OTU_240</td><td>-0.073</td><td>0.089</td><td>0.017</td><td>0.008521</td><td>0.085292</td><td>-0.083</td><td>0.006</td><td>7.82E-07</td><td>1.99E-05</td></tr>
<tr><td>Aggregatibacter_segnis|sp._oral_taxon_513_OTU_165</td><td>-0.136</td><td>0.177</td><td>0.041</td><td>0.000709</td><td>0.012369</td><td>-0.100</td><td>0.078</td><td>0.201136</td><td>0.490373</td></tr>
<tr><td>Porphyromonas_pasteri_OTU_180</td><td>-0.186</td><td>0.384</td><td>0.199</td><td>0.129418</td><td>0.45721</td><td>-0.242</td><td>0.142</td><td>0.005261</td><td>0.048099</td></tr>
<tr><td>Staphylococcus_epidermidis_OTU_404</td><td>-0.370</td><td>0.374</td><td>0.004</td><td>0</td><td>0</td><td>-0.367</td><td>0.007</td><td>1.05E-10</td><td>6.24E-09</td></tr>
<tr><td>Streptococcus_dentisani|mitis_OTU_143</td><td>-0.585</td><td>1.096</td><td>0.511</td><td>0.000284</td><td>0.00692</td><td>-0.021</td><td>1.075</td><td>0.944756</td><td>0.923424</td></tr>
<tr><td>Neisseria_elongata_OTU_72</td><td>-1.194</td><td>2.006</td><td>0.812</td><td>0.003506</td><td>0.044076</td><td>-0.887</td><td>1.119</td><td>0.105215</td><td>0.34686</td></tr>
<tr><td>Fusobacterium_nucleatum_ss_polymorphum_OTU_139</td><td>-1.074</td><td>3.143</td><td>2.069</td><td>0.013295</td><td>0.114067</td><td>-1.385</td><td>1.757</td><td>0.000679</td><td>0.008234</td></tr>
</table>

Table S2 - Changes in mean relative abundance observed for the significantly different taxa following use of the control toothpaste at 2 and 6 weeks compared to baseline (p<0.05 and q<0.05).

Taxa	Change in mean relative abundance baseline - 2 weeks (%)	Mean relative abundance at baseline (%)	Mean relative abundance after 2 weeks (%)	p values	q values	Change in mean relative abundance baseline - 6 weeks (%)	Mean relative abundance after 6 weeks (%)	p values	q values
Staphylococcus_epidermidis_OTU_404	0.0091	0.001	0.010	8.57E-08	6.18E-06	0.003	0.004	0.007922	0.115601
Treponema_denticola_OTU_453	-0.0251	0.029	0.004	0.001643	0.047391	-0.012	0.017	0.50162	0.907451
Filifactor_alocis_OTU_425	-0.0017	0.007	0.005	0.460093	0.96431	0.031	0.038	0.000911	0.021241
Veillonella_sp._oral_taxon_780_OTU_291	0.0005	0.006	0.006	0.940854	1	-0.005	0.001	0.000123	0.003398
Granulicatella_elegans_OTU_266	0.0236	0.032	0.055	0.483451	0.965249	-0.026	0.005	4.86E-05	0.001562
Haemophilus_haemolyticus_OTU_269	-0.0225	0.033	0.011	0.045405	0.363872	-0.027	0.006	0.000917	0.021241
Haemophilus_parahaemolyticus_OTU_440	-0.0141	0.035	0.020	0.504652	0.965252	-0.032	0.003	2.22E-07	9.16E-06
Aggregatibacter_aphrophilus_OTU_350	-0.0353	0.048	0.000	0.028673	0.298221	-0.046	0.002	6.16E-10	5.94E-08

Table S3 - Bacterial pathways with signficiant differences between the zinc and control toothpaste compared at baseline, 2 and 6 weeks using LefSe, the values are the LDA values from the analysis, green are associated with the zinc toothpaste and red with the control

	Zinc			Control		
**Pathway**	**Baseline**	**2wks**	**6wks**	**Baseline**	**2wks**	**6wks**
Arginineandprolinemetabolism_ko00330			2.376667			
Biotinmetabolism_ko00780		2.15328	2.203195			
Carbonfixationpathwaysinprokaryotes_ko00720		2.265513	2.381425			
Citratecycle_TCAcycle__ko00020			2.177695			
Cysteineandmethioninemetabolism_ko00270			2.393635			
Folatebiosynthesis_ko00790			2.012742			
Glyoxylateanddicarboxylatemetabolism_ko00630			2.38831			
Nitrogenmetabolism_ko00910			2.750432			
Nitrotoluenedegradation_ko00633			2.205684			
Pertussis_ko05133	2.562744		2.684277			
Phenylalanine_tyrosineandtryptophanbiosynthesis_ko00400			2.374109			
Porphyrinandchlorophyllmetabolism_ko00860		2.809919	2.925532			
Pyruvatemetabolism_ko00620			2.171136			
Ubiquinoneandotherterpenoid_quinonebiosynthesis_ko00130		2.195491	2.25654			
Valine_leucineandisoleucinebiosynthesis_ko00290			2.107305			
Aminosugarandnucleotidesugarmetabolism_ko00520						2.60904
D_Alaninemetabolism_ko00473						2.154925
Fructoseandmannosemetabolism_ko00051						2.583552
Galactosemetabolism_ko00052						2.625874
Glycerophospholipidmetabolism_ko00564						2.272171
Glycolysis_Gluconeogenesis_ko00010					2.486691	2.570259
Insulinsignalingpathway_ko04910						2.001751
Nucleotideexcisionrepair_ko03420				2.093471		
Pentoseandglucuronateinterconversions_ko00040					2.340842	2.383589
Phosphotransferasesystem_PTS__ko02060						2.807879
Sphingolipidmetabolism_ko00600						2.055858
Starchandsucrosemetabolism_ko00500						2.860972

Table S4 - Bacterial pathways with signficiant differences between timepoints Base-2wks and Base-6wks for the zinc and control toothpaste using LefSe, the values are the LDA values from the analysis coloured by product (orange and green for zinc toothpaste, yellow and blue for control toothpaste) and timepoint (orange and yellow for baseline, green and blue for 2 or 6 wks).

	**Zinc**				**Control**			
	**Base-2wks**		**Base-6wks**		**Base-2wks**		**Base-6wks**	
**Pathway**	**Base**	**2wks**	**Base**	**6wks**	**Base**	**2wks**	**Base**	**6wks**
ABCtransporters_ko02010				2.97762				
Alanine_aspartateandglutamatemetabolism_ko00250		2.098065		2.220879				
Aminoacyl_tRNAbiosynthesis_ko00970		2.717592		2.755099				2.575509
Aminobenzoatedegradation_ko00627	2.246641		2.347817		2.208723			
Aminosugarandnucleotidesugarmetabolism_ko00520			2.427373					
Arginineandprolinemetabolism_ko00330				2.285717				
Ascorbateandaldaratemetabolism_ko00053			2.04415					
Bacterialchemotaxis_ko02030	2.219643							
Benzoatedegradation_ko00362	2.022122		2.130042					
Biosynthesisof12__14_and16_memberedmacrolides_ko00522			2.090628					
Biosynthesisofansamycins_ko01051			2.183434					
Biotinmetabolism_ko00780		2.146218		2.22397				
Bisphenoldegradation_ko00363			2.06499					
Butanoatemetabolism_ko00650	2.271076		2.322616					
Carbonfixationpathwaysinprokaryotes_ko00720		2.416135		2.456932				
Citratecycle_TCAcycle__ko00020		2.157195		2.232848				
Cysteineandmethioninemetabolism_ko00270		2.558129		2.570685				
Fattyacidbiosynthesis_ko00061					2.246673			
Fattyacidmetabolism_ko00071	2.170191		2.303981				2.157757	
Folatebiosynthesis_ko00790		2.160686		2.220927				
Fructoseandmannosemetabolism_ko00051	2.558557		2.648927					
Galactosemetabolism_ko00052	2.556921		2.627005					
Geranioldegradation_ko00281	2.192017		2.339693		2.257095		2.246686	
Glycerolipidmetabolism_ko00561	2.117732		2.320961					
Glycerophospholipidmetabolism_ko00564	2.4583		2.451224					
Glycolysis_Gluconeogenesis_ko00010	2.485846		2.568226					
Histidinemetabolism_ko00340		2.008519		2.016263				
Homologousrecombination_ko03440		2.082128						
Limoneneandpinenedegradation_ko00903			2.114043		2.07436			
Lipopolysaccharidebiosynthesis_ko00540		2.493845						
Lysinebiosynthesis_ko00300		2.111039		2.162122				
Lysinedegradation_ko00310	2.236201		2.265194		2.147815			
MetabolismofxenobioticsbycytochromeP450_ko00980							2.005211	
Methanemetabolism_ko00680		2.286446						
Nitrogenmetabolism_ko00910		2.796681		2.847563				
Nitrotoluenedegradation_ko00633		2.238315						
Nonribosomalpeptidestructures_ko01054	2.189519		2.309273				2.271498	
Nucleotideexcisionrepair_ko03420		2.276798						
PantothenateandCoAbiosynthesis_ko00770				2.026059				
Pentoseandglucuronateinterconversions_ko00040	2.387231		2.471376					
Phenylalanine_tyrosineandtryptophanbiosynthesis_ko00400		2.596714						
Phosphotransferasesystem_PTS__ko02060			2.809412					
Porphyrinandchlorophyllmetabolism_ko00860		3.152023		3.168803				
Purinemetabolism_ko00230		2.800878		2.826018				2.551414
Pyrimidinemetabolism_ko00240		2.456479		2.512123				2.424709
Pyruvatemetabolism_ko00620				2.07894				
Ribosome_ko03010		2.533469		2.573737				2.378935
RNAdegradation_ko03018				2.148655				
Selenocompoundmetabolism_ko00450		2.227814		2.252188				
Sphingolipidmetabolism_ko00600	2.02905		2.073594					
Starchandsucrosemetabolism_ko00500	2.868128		2.934203					
Steroiddegradation_ko00984			2.062861					
Terpenoidbackbonebiosynthesis_ko00900		2.071899		2.084648				
Thiaminemetabolism_ko00730		2.509824		2.549548				
Two_componentsystem_ko02020	3.025789		2.752958					
Tyrosinemetabolism_ko00350	2.032017		2.086177					
Valine_leucineandisoleucinebiosynthesis_ko00290		2.13824		2.13137				
Valine_leucineandisoleucinedegradation_ko00280			2.152442		2.258636			
Vibriocholeraepathogeniccycle_ko05111	2.278024		2.165451					

Oral Metataxonomic Bacterial Processing Pipeline (Illumina) report

Raw sequencing reads were processed simultaneously as follows. PCR primers used for initial 16S rRNA gene amplification were removed from each fragment using Cutadapt [1] version 1.14 due to the presence of degenerate bases that may impact downstream taxonomic assessment. Sickle version 1.33 [2] was used to quality trim DNA reads using a minimum quality value of 28. Reads less than 100 bp following quality trimming were discarded. If a single read was discarded during this process its read pair was also discarded. Reads passing filtering were merged using Pandaseq version 2.9 [3] to generate overlapping contigs with a minimum overlap of 20 bp and a minimum amplicon length of 200 bp. The resulting overlapped reads were de-replicated using Vsearch version v1.9.6 linux x86 64 [4] and searched against a BLAST database composed of the HOMD, HOMD extended and Greenegenes sequences (HOMDEXTGG) described in [5]. Taxonomic classification was then performed as previously described [6] at 99 % identity across 98 % of the read length. Reads not classified by this process were discarded. This process resulted in 908 taxonomically classified OTUs. The resulting classification table and associated representative sequences, selected as the most abundant sequence for each classified taxa, were used as inputs for QIIME [7] (Quantitate Insights into Microbial Ecology) version 1.9.1. To perform the functional predictions, the OTUs were assigned taxonomies using QIIME's assign taxonomy script, with the default settings and using SILVA release 123 as the reference database. Samples with less than 1000 counts were filtered from the resulting OTU table, which was then rarefied to the 5th percentile to account for variable sequencing depths. OTUs representing less than 0.5 % of the total sequence count were then removed. The OTU table was then analysed with Tax4Fun [8] version 0.3.1, using the default settings and metabolic profiles calculated against SILVA release 123.

References

[1] Martin M. Cutadapt Removes Adapter Sequences From High-Throughput Sequencing Reads EMBnet.journal, 2011 17:10-12 doi:http://dx.doi.org/10.14806/ej.17.1.200 http://cutadapt.readthedocs.io/en/stable/

[2] Joshi NA, Fass JN. (2011). Sickle: A sliding-window, adaptive, quality-based trimming tool for FastQ files https://github.com/najoshi/sickle

[3] Masella AP, Bartram AK, Truszkowski JM, Brown DG and Neufeld JD PANDAseq, paired-end assembler for illumina sequences, BMC Bioinformatics, 2012, vol 13, pp. 31

[4] Vsearch: Flouri T, Quince C, and Nicholos B https://github.com/torognes/vsearch

[5] Al-Hebshi NN, Nasher AT, Idris AM, Chen T. Robust species taxonomy assignment algorithm for 16S rRNA NGS reads: application to oral carcinoma samples 2015;7:10.3402/jom.v7.28934. doi:10.3402/jom.v7.28934.

[6] Chen T et. al., The Human Oral Microbiome Database: a web accessible resource for investigating oral microbe taxonomic and genomic information, Database (Oxford), 2010, baq013, doi:10.1093/database/baq013. http://www.homd.org

[7] Caporaso JG, Kuczynski J, Stombaugh J, et al. QIIME allows analysis of high-throughput community sequencing data, Nature Methods, 2010; 7(5):335-336 doi:10.1038/nmeth.f.303

[8] Aβhauer, K.P. et al. Tax4Fun: predicting functional profiles from metagenomic 16S rRNA data Bioinformatics, 2015, 31:2882–2884. http://tax4fun.gobics.de/

Supplementary information

Shotgun Metagenomics Pipeline Report

Metatranscriptomic reads were processed simultaneously as follows. FastQC version v0.11.5 was used to analyse the quality of each sample [1]. Only samples with a gc content between 25 and 75 percent (inclusive) and an average base quality of at least 20 were analysed further. Trimmomatic version 0.36 [2] was used to quality trim DNA reads using a minimum average quality value of 28 over a sliding window of length 100. Reads less than 100 bp following quality trimming were discarded. If a single read was discarded during this process its read pair was also discarded. Reads passing this filter were then run through Kneaddata v0.5.1 [3] to remove human sequences. Reads were aligned to Kneaddata's default human database using Bowtie2 version 2.2.9, and any aligned reads were discarded from the dataset. Unmapped reads were then classified taxonomically and functionally. Taxonomic classification was performed using Kraken version 1.0 [4] against an in-house generated database based on HOMD (Human Oral Microbial Database) [5], with a custom Neisseria flava species added. The Kraken classifications were then analysed by Bracken version 0.1 [6] to produce species-level abundance estimates. To minimise false positives during Bracken estimation, a minimum of 10 reads were required for a classification to be subject to re-estimation. Taxonomic output was visualised using the GraPhlAn (version 1.1) and Krona (version v2.7) visualisation tools for shotgun metagenomics data [7], [8]. Functional classification was performed with HUMAnN2 version 0.9.3 [9], using the default databases for pathway coverage and abundance, and UniRef90 for the gene families. HUMAnN2 used MetaPhlAn2 version 2.6.0 [10] for an initial taxonomic assignment, Bowtie2 version 2.2.9 for nucleotide alignments and DIAMOND version 0.8.22 [11] for translated read alignments. The gene family results from HUMAnN2 were mapped to a GO slim designed specifically for metagenomics (http://www.geneontology.org/GO slims/goslim metagenomics.obo).

References

[1] FastQC: Andrews S http://www.bioinformatics.babraham.ac.uk/projects/fastqc/

[2] Bolger AM, Lohse M, Usadel B. Trimmomatic: a flexible trimmer for Illumina sequence data Bioinformatics 2014 30:2114-20
doi:10.1093/bioinformatics/btu170 http://www.usadellab.org/cms/?page=trimmomatic

[3] KneadData: Shi A et.al. http://huttenhower.org/kneaddata

[4] Wood DE, Salburg SL. Kraken: ultrafast metagenomic sequence classification using exact alignments Genome Biology 2014 15:R46
doi:10.1186/gb-2014-15-3-r46 http://ccb.jhu.edu/software/kraken/

[5] Chen T et. al., The Human Oral Microbiome Database: a web accessible resource for investigating oral microbe taxonomic and genomic information, Database (Oxford), 2010, baq013, doi:10.1093/database/baq013. http://www.homd.org

[6] Lu J et.al. Bracken: Estimating species abundance in metagenomics data. bioRxiv doi: http://dx.doi.org/10.1101/051813 http://biorxiv.org/content/early/2016/05/05/051813

[7] Graphplan: Segata N. https://huttenhower.sph.harvard.edu/graphlan

[8] Ondov BD, Bergman NH, Phillippy AM. Interactive metagenomic visualization in a Web browser BMC Bioinformatics. 2011 12:385 doi:10.1186/1471-2105-12-385

[9] HUMAnN2: http://huttenhower.sph.harvard.edu/humann2

[10] Truong et.al. MetaPhlAn2 for enhanced metagenomic taxonomic profiling. Nature Methods. 2015 12:902-3. doi:10.1038/nmeth.3589

[11] Buchfink B, Xie C, Huson DH. Fast and sensitive protein alignment using DIAMOND Nature Methods 2015 12:59–60 doi:10.1038/nmeth.3176

Any changes to trial outcomes after the trial commenced, with reasons.

Samples were to be collected by a single clinician across the study to ensure consistency in sample collection. Due to illness, the study clinician was unavailable for sample collection at week 12. Samples were collected by multiple other clinicians. Initial data analysis indicated that samples at week 12 were not consistent with weeks 0, 2 and 6 [increased levels of DNA] and were subsequently removed from analysis.
